# Comparative Transcriptomics Reveals Novel and Differential Long-Noncoding RNA Responses Underlying Interferon-Mediated Antiviral Regulation in Porcine Alveolar Macrophages

**DOI:** 10.3390/pathogens15010035

**Published:** 2025-12-26

**Authors:** Jiuyi Li, Oluwaseun Adeyemi, Laura C. Miller, Yongming Sang

**Affiliations:** 1 Department of Food and Animal Sciences, College of Agriculture, Tennessee State University, Nashville, TN 37209, USA; jli4@tnstate.edu (J.L.); oadeyemi@my.tnstate.edu (O.A.); 2 Diagnostic Medicine and Pathobiology, College of Veterinary Medicine, Kansas State University, 1800 Denison Ave., Manhattan, KS 66506, USA; lauramiller@ksu.edu

**Keywords:** porcine reproductive and respiratory syndrome, PRRS virus, alveolar macrophage, long non-coding RNA, interferon

## Abstract

Porcine reproductive and respiratory syndrome virus (PRRSV) is a major threat to the global swine industry. Long non-coding RNAs (lncRNAs) are emerging as crucial regulators of antiviral immunity, but their roles in porcine alveolar macrophages (PAMs)—the primary target of PRRSV—remain poorly characterized. This study presents a genome-wide analysis of lncRNA expression in PAMs stimulated with a PRRS modified live virus (MLV) vaccine and two type I interferons, IFN-α1 and IFN-ω5. Whole-transcriptome sequencing identified over 2000 differentially expressed lncRNAs, with IFN-ω5 inducing the most extensive transcriptional reprogramming. Weighted gene co-expression network analysis (WGCNA) revealed interferon-specific lncRNA-mRNA modules, and functional enrichment showed these lncRNAs are involved in key immune and metabolic pathways, including chemokine signaling, MAPK, and mTOR. Our findings establish a comprehensive landscape of lncRNA regulation in PAMs, highlighting their role in fine-tuning the antiviral responses and suggesting novel targets for interferon-based antiviral interventions against PRRSV.

## 1. Introduction

Porcine reproductive and respiratory syndrome (PRRS) is one of the most economically important diseases in swine worldwide. The disease is characterized by reproductive disorders in sows and respiratory diseases in growing pigs, imposing a substantial economic burden on the global pig farming industry [1]. The causative agent, PRRS virus (PRRSV), is an enveloped, single-stranded RNA virus in the *Arteriviridae* family, sharing some genomic structural features with coronaviruses [2,3]. PRRSV exhibits strict cellular tropism; the primary target cells are porcine alveolar macrophages (PAMs). Replication in infected PAMs alters their morphology and function. As the frontline of the immune system within the alveolar space, PAMs play a crucial role in initiating and regulating immune responses. Their surface receptors are involved in controlling phagocytosis and efferocytosis, maintaining the ability to clear apoptotic cells from the alveoli. Beyond their anti-inflammatory functions, PAMs also release various cytokines and immunoregulatory mediators, including CD200, TGF-β, CD172a, GM-CSF, M-CSF, and IL-10 [4,5]. A recent study reported that PAMs express at least 13 antiviral proteins capable of inhibiting viral replication, underscoring their crucial role in the innate immune defense against PRRSV infection [6]. This finding aligns with evidence that PAMs can acquire a form of “trained immunity,” an adaptive-like state driven by epigenetic reprogramming that enhances their responsiveness to subsequent viral challenges [7].

Long noncoding RNAs (lncRNAs) are members of the noncoding RNA (ncRNA) family and are typically longer than 200 nucleotides. They are distributed in both the nucleus and the cytoplasm [8]. Like other ncRNAs, such as microRNAs and circular RNAs, lncRNAs lack protein-coding capacity. Instead, they play essential regulatory roles in various cellular processes by modulating gene expression at the transcriptional, post-transcriptional, and epigenetic levels [9]. At the mechanistic level, lncRNAs can act in cis, regulating nearby protein-coding genes within the same genomic locus, or in trans, modulating distant targets, often on other chromosomes. In many cases, lncRNAs also serve as proxy signals for cis-regulatory elements, marking enhancer- or promoter-like regions that influence local gene expression. Distinguishing these *cis-* and *trans*-regulatory modes is critical for interpreting lncRNA–mRNA associations from high-throughput transcriptomic data [9]. Several studies have further emphasized the regulatory role of lncRNAs in shaping immune memory and cellular defense mechanisms. For instance, Flores-Concha et al. identified specific lncRNAs as key modulators involved in the training of immune responses, suggesting that these molecules contribute to the establishment of enhanced innate immunity [10]. Similarly, Wang et al. reported that lncRNA LOC103222771 may act as an upstream regulator of CLDN4, a critical component of tight junctions responsible for maintaining the paracellular barrier and controlling molecular flux between epithelial cells [11]. These findings indicate the multifaceted roles of lncRNAs in both immune regulation and epithelial integrity, processes that are essential for effective antiviral defense [12].

Recent studies have also analyzed the interactions between lncRNAs and gene expression in the context of PRRSV infection [13,14,15,16]. However, despite increasing recognition of the importance of lncRNAs in immune regulation, their global expression landscape and functional roles in PAMs during PRRSV infection or interferon stimulation remain largely undefined. No comprehensive analysis has yet explored how lncRNAs coordinate *cis*- and *trans*-regulatory networks or participate in interferon-mediated transcriptional control in this system [17]. Addressing these knowledge gaps is crucial for elucidating the molecular mechanisms underlying host–virus interactions and may provide new avenues for lncRNA-based antiviral interventions and the optimization of PRRSV vaccine strategies.

The present study aimed to investigate the genome-wide landscape of lncRNA expression in PAMs following stimulation with a PRRS modified live virus (MLV) vaccine and two type I interferon subtypes, IFN-α1 and IFN-ω5. To address this objective, whole-transcriptome sequencing was performed to comprehensively profile differentially expressed lncRNAs under each antiviral treatment. To our knowledge, this work represents the first large-scale analysis of interferon-induced lncRNA responses within the context of PRRSV infection, extending our previous findings on miRNA and circRNA regulation in the same cellular model. Across all treatment groups, more than 2000 lncRNAs exhibited significant differential expression. Functional enrichment through Gene Ontology (GO) and KEGG pathway analyses revealed that these lncRNA-associated genes are primarily involved in immune modulation, antiviral defense, and cellular signaling. Furthermore, weighted gene co-expression network analysis (WGCNA) was employed to elucidate the interactions between lncRNAs and their potential cis- and trans-acting target genes, providing insights into their network-level regulatory roles.

## 2. Materials and Method

### 2.1. Ethics Statement and Animal Cell Sources

No live animal experiments were performed in the present study. Porcine primary cells were derived from previously established cryopreserved stock maintained in our laboratory [18]. All recombinant DNA and animal-related protocols were reviewed and authorized by the Institutional Biosafety Committee and the Institutional Animal Care and Use Committee (IBC#1732 and IACUC#5349, respectively). Porcine alveolar macrophages were obtained through bronchoalveolar lavage of lungs collected from six outbred piglets approximately five weeks of age. For each treatment condition, experiments were performed in three independent biological replicates. For RNA-seq library preparation, cells from the three replicates were pooled within each treatment group prior to RNA extraction (one pooled library per group). Each lavage was conducted using 300 mL of 10 mM phosphate-buffered saline (PBS, pH 7.4). Within four hours of sampling, PAMs were recovered by centrifugation at 400× *g* for 15 min and enriched by plastic adherence. The resulting cell preparations were either used immediately for downstream analyses or preserved in Recovery™ Cell Culture Freezing Medium (Invitrogen, Carlsbad, CA, USA) and stored in liquid nitrogen. For PRRSV propagation and antiviral testing, the MARC-145 cell line (African green monkey kidney; ATCC) was maintained according to the supplier’s specifications or as previously reported [18].

### 2.2. Virus Infection and IFN Treatment

Porcine alveolar macrophages were plated in 6-well culture dishes and maintained in RPMI medium supplemented with 10% fetal bovine serum (FBS; Thermo Fisher Scientific, Waltham, MA, USA) and 1× penicillin-streptomycin. For the PRRSV condition, a single defined PRRS MLV vaccine strain (Ingelvac PRRS MLV; Boehringer Ingelheim Vetmedica, Duluth, GA, USA) was used as the standardized viral stimulus in this experimental system. Four experimental conditions were established, each performed with three biological replicates: (i) unstimulated control, (ii) PRRS MLV vaccine strain, (iii) recombinant porcine IFN-α1, and (iv) IFN-ω5 (Kingfisher Biotech, Saint Paul, MN, USA) administered at a final dose of 20 ng/mL for 5 h as optimized previously to capture early, direct transcriptional responses [18]. PRRSV inoculation was conducted at a multiplicity of infection (MOI) of 0.1 for 5 h. Following incubation, residual virus was removed by washing the cells twice with fresh medium prior to downstream RNA and protein extraction [18]. To generate a baseline control response, bovine serum albumin (BSA; Thermo Fisher Scientific, Waltham, MA, USA) was added to the control cultures at the same concentration used for IFN treatment. To verify the biological responsiveness of PAMs to stimulation, induction of representative interferon-stimulated genes (ISGs), including IFNARs and IRF family members, was confirmed by RT-PCR following previously established procedures [18].

### 2.3. RNA Transcriptomic Library Construction

For each treatment condition, approximately 2.5 × 10^7^ PAMs from three experimental replicates were processed for total RNA extraction using a column-based RNA/DNA/protein purification system (Norgen Biotek, Thorold, ON, Canada). RNA purity and concentration were evaluated using a NanoPhotometer^®^ (IMPLEN, Westlake Village, CA, USA) and a Qubit^®^ 2.0 Fluorometer equipped with the Qubit^®^ RNA Assay Kit (Life Technologies, Carlsbad, CA, USA). Samples with an A260/A280 ratio above 1.8 and an RNA integrity number (RIN) of at least 7.0 were considered acceptable for downstream library generation. A total of 5 μg RNA from each qualified sample was subjected to depletion of ribosomal RNA using the Ribo-Zero™ rRNA Removal Kit (Epicentre, Madison, WI, USA), followed by ethanol precipitation. Sequencing libraries were generated using the NEBNext^®^ Ultra™ Directional RNA Library Prep Kit for Illumina^®^ (New England Biolabs, MA, USA). Briefly, first-strand cDNA synthesis was carried out using random hexamer primers, followed by second-strand synthesis incorporating dUTP to maintain strand specificity. Subsequent steps included end repair, 3′ adenylation, and adaptor ligation. Target fragment lengths (~150–200 bp) were enriched using AMPure XP bead selection, treated with USER enzyme (3 μL; NEB), and subjected to limited-cycle PCR amplification. Final libraries were cleaned using AMPure XP beads, and quality was assessed on an Agilent Bioanalyzer 2100 [18].

### 2.4. RNA Transcriptomic Analysis

As illustrated in Figure S1 (Supplemental Material), raw sequencing reads were pre-processed to remove adaptor sequences, poly-N stretches, and low-quality reads, yielding high-quality clean data. Quality metrics, including Q20, Q30, and GC content, were calculated to assess sequencing accuracy. The *Sus scrofa* reference genome and corresponding annotation files were obtained from Ensembl (release 111), and genome indexing was conducted using HISAT2 (v2.0.4). Paired-end clean reads were aligned to the reference genome using HISAT2 with strand-specific settings, and transcript assembly was performed using StringTie (v1.3.3b). All assembled transcripts were merged to construct a comprehensive, non-redundant transcriptome reference [18].

Candidate lncRNAs were screened through a multi-step filtering process, removing transcripts shorter than 200 nucleotides or containing fewer than two exons, followed by the exclusion of known protein-coding and small RNAs. The coding potential of the remaining transcripts was evaluated using CPC2, CNCI, and PFAM, and those predicted as non-coding by all tools were retained as high-confidence lncRNAs. Expression levels were quantified and normalized as FPKM, and differential expression analysis was conducted using DESeq2 (v1.24.0) [18,19,20].

To further explore potential functional relationships among lncRNAs and their associated genes, weighted gene co-expression network analysis (WGCNA) was applied [21]. This approach identified co-expression modules and revealed module–trait correlations associated with vaccine and interferon treatments, providing insights into lncRNA-mediated regulatory networks during antiviral immune responses. Functional enrichment analyses of lncRNA-targeted genes were performed using GOseq for Gene Ontology and KEGG pathway annotation [22]. All software and parameters used are listed in Table S1 (Supplemental Material), and raw sequencing data have been deposited in the NCBI Short Read Archive under BioProject accession number PRJNA882823.

### 2.5. Statistical Analysis

Statistical analyses were performed using the SAS-v9.4 software package. One-way analysis of variance (ANOVA) followed by Tukey’s post hoc test, as well as a two-sample F-test, was used to evaluate differences between samples/treatments. A *p*-value < 0.05 was considered statistically significant [23].

## 3. Results

### 3.1. LncRNA Compositions and Identification Across the PAM Samples

In our previous work, we characterized the differential expression profiles of microRNAs and circRNAs during PRRSV infection, contributing to the limited transcriptomic landscape available for the PRRSV–swine interaction model [5,18]. Building on these insights and extending the scope of ncRNA regulation, the present study specifically investigates the lncRNA-mediated transcriptional responses in porcine primary macrophages following stimulation with a PRRS MLV vaccine and two porcine interferon subtypes, IFN-α1 and IFN-ω5.

As detailed in Supplementary Excel Table S2, high-throughput sequencing yielded between 83.08 and 91.86 million raw reads across the four groups. Following adaptor trimming and the removal of low-quality reads, the number of clean reads ranged from 81.70 to 90.25 million, resulting in 12.3–13.5 Gb of high-quality bases available for downstream analysis. All sequencing libraries exhibited consistently low base-calling error rates of 0.03%, indicating high technical accuracy. Quality score assessment further revealed that Q20 values were above 97% and Q30 values exceeded 92% in every sample, confirming that the vast majority of bases had a low probability of sequencing error. The GC content ranged from 52.20% to 53.74%, which falls within the expected range for the porcine transcriptome and indicates no obvious GC bias during library construction.

The majority of mapped reads were assigned to protein-coding transcripts, accounting for around 73–76% of total aligned reads across all samples (Figure S2), indicating that the dataset captures the primary transcriptional output of PAMs under antiviral stimulation. A small but consistent fraction (~1.5–1.9%) of reads was annotated as lncRNA, representing a sufficient sequencing depth for confident lncRNA detection and expression quantification. Reads mapping to rRNA, tRNA, and pseudogene classes remained below 0.2%, suggesting minimal contamination from highly abundant housekeeping RNAs. The proportion of ribozyme-associated reads (~1.24–1.35%) remained stable across samples, further indicating technical consistency among sequencing libraries. Table 1 summarizes the alignment performance of clean reads from samples P1–P4. All libraries generated between 81 and 90 million total reads, with high overall mapping rates (86–89%), indicating strong compatibility with the reference genome. The majority of reads were uniquely mapped (>81%), while multi-mapped reads remained low (~4–5%), reflecting minimal ambiguity and high library specificity. Strand distribution was balanced, and more than 80% of reads were mapped in proper pairs, demonstrating good sequencing quality and alignment accuracy. Additionally, splice reads accounted for approximately 30% of mapped reads across all samples, confirming effective capture of exon–exon junctions required for reliable transcript reconstruction and lncRNA identification. To complement these alignment metrics, the genomic distribution of mapped reads provides further confirmation of dataset uniformity (Figure S3). Read density profiles along each chromosome were highly consistent among P1–P4, with no evidence of localized dropouts or coverage gaps. Coverage patterns corresponded closely to chromosome size, with larger chromosomes (e.g., 1, 13, 6, 2) exhibiting proportionally higher read densities. The smooth and continuous chromosomal distribution indicates even library complexity and robust sequencing performance across samples. Together, these results demonstrate that the RNA-seq datasets possess sufficient depth, accuracy, and uniformity to support downstream differential expression, lncRNA profiling, and co-expression network analyses.

### 3.2. Filtering of Candidate lncRNAs

**Figure 1:**
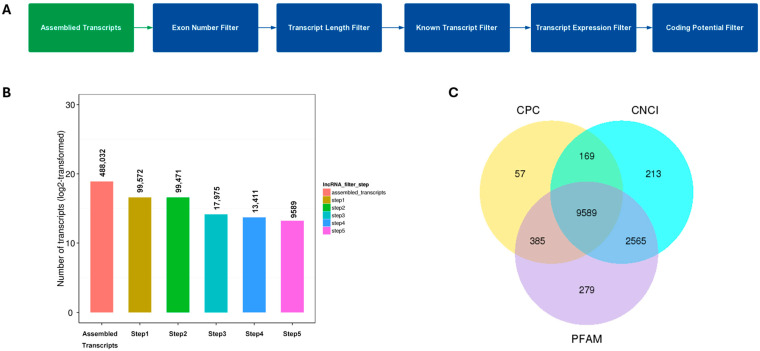
Multi-step filtering pipeline and identification of high-confidence lncRNAs. (**A**) Overview of the sequential filtering workflow applied to assembled transcripts for lncRNA identification. The process included exon number, transcript length, known transcript, expression, and coding potential filters to progressively refine the dataset and eliminate transcripts with potential protein-coding features. (**B**) Bar chart showing the number of transcripts (log_2_-transformed) remaining after each filtering step. Starting from 488,032 assembled transcripts, the stepwise filtration reduced the dataset to 9589 high-confidence lncRNA candidates. The most substantial decreases occurred after the exon number and expression filters, demonstrating their strong impact in removing low-confidence or single-exon transcripts. (**C**) Venn diagram displaying the overlap of transcripts predicted as non-coding by three independent coding potential prediction tools—CPC, CNCI, and PFAM. The intersection of all three methods (9589 transcripts) represents the final set of putative lncRNAs consistently classified as non-coding by all prediction algorithms, ensuring high confidence and minimal false positives for downstream analyses.

To identify lncRNAs longer than 200 nucleotides, a multi-step filtering pipeline was applied. The filtering process included an exon number filter, transcript length filter, known transcript filter, transcript expression filter, and coding potential filter (Figure 1A). These sequential steps were designed to remove sequencing noise, ensure that transcripts met the length criterion, exclude known protein-coding genes, retain transcripts with reliable expression levels, and confirm that candidate transcripts lacked protein-coding potential. Because lncRNAs are structurally diverse and often difficult to distinguish from protein-coding transcripts, this comprehensive filtering strategy effectively eliminates all possible coding sequences, yielding a high-confidence set of putative lncRNAs.

From Figure 1B, starting with 488,032 assembled transcripts, the successive application of exon number, transcript length, known transcript, expression, and coding potential filters ultimately yielded 9589 high-confidence lncRNA candidates. The most substantial reductions occurred after the exon number and expression filters, reflecting the effective removal of low-confidence, single-exon, or weakly expressed transcripts. This progressive decrease demonstrates the efficiency and stringency of the filtering pipeline in refining the dataset into a reliable set of putative lncRNAs for downstream analyses.

Figure 1C further illustrates the results of coding potential prediction, presented as a Venn diagram comparing outputs from CPC, CNCI, and PFAM analyses. Each circle represents transcripts predicted as non-coding by a single tool, while the overlapping regions indicate transcripts consistently classified as non-coding by multiple methods. Transcripts identified by any of the prediction software as having coding potential were classified as TUCPs (Transcripts of Uncertain Coding Potential), whereas those consistently predicted as noncoding by all software were retained as lncRNAs. A total of 9589 transcripts were simultaneously identified as non-coding by all three tools, constituting the final set of high-confidence lncRNAs. This intersection-based approach minimizes false positives and ensures that only transcripts consistently validated across multiple algorithms are retained for subsequent analyses.

**Figure 2:**
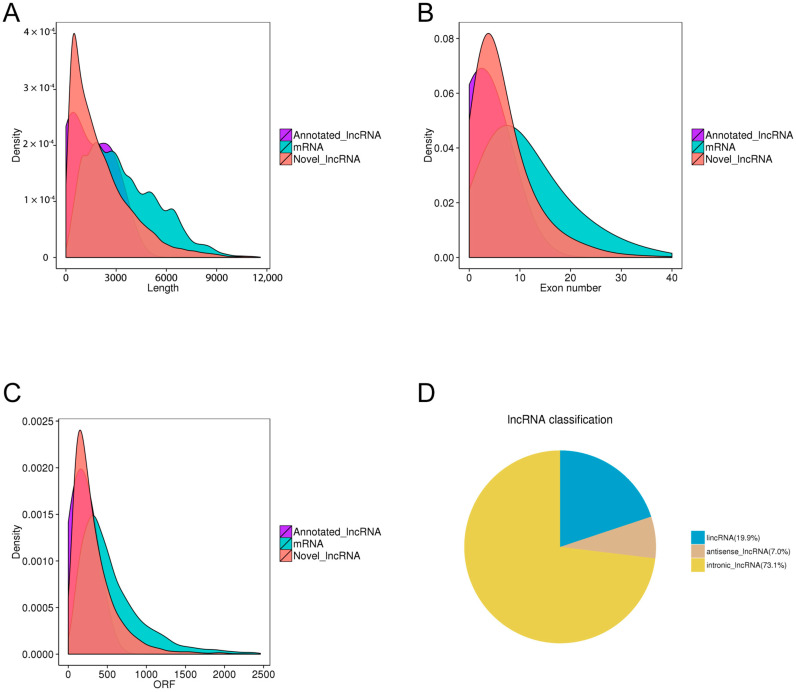
Structural features and classification of identified lncRNAs in PAMs. (**A**–**C**) Density distributions of transcript length, exon number, and open reading frame (ORF) length comparing annotated lncRNAs, novel lncRNAs, and mRNAs. Both annotated and novel lncRNAs exhibited shorter transcript and ORF lengths with fewer exons than mRNAs, consistent with typical non-coding RNA characteristics. (**D**) Genomic classification of lncRNAs showing that intronic lncRNAs accounted for the majority (73.1%), followed by intergenic (lincRNAs, 19.9%) and antisense lncRNAs (7.0%).

To validate the structural characteristics of the predicted lncRNAs, transcript length, exon number, and open reading frame (ORF) length were compared with those of mRNAs. The ORFs of lncRNAs were predicted using EMBOSS (getorf), while ORF lengths of mRNAs were obtained from genome annotations. As shown in Figure 2A–C, both annotated and novel lncRNAs displayed shorter transcript lengths, fewer exons, and markedly shorter ORFs compared with mRNAs, which is consistent with the structural characteristics typical of non-coding RNAs. These patterns validate that the predicted lncRNAs are distinct from protein-coding transcripts. Furthermore, the classification of lncRNAs based on their genomic positions relative to known protein-coding genes revealed that intronic lncRNAs represented the largest group (73.1%), followed by intronic lncRNAs (lincRNA at 19.9%) and antisense lncRNAs (7.0%) (Figure 2D). This distribution reflects the diverse transcriptional origins and potential regulatory complexity of lncRNAs in porcine alveolar macrophages.

### 3.3. Quantification of lncRNAs, mRNA and TUCP

**Figure 3:**
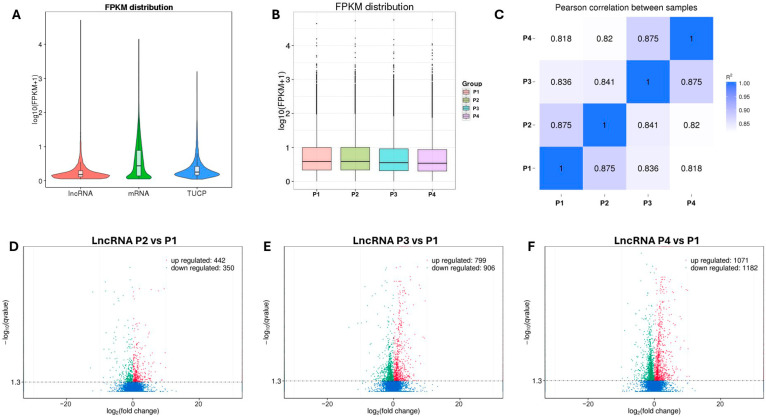
Global lncRNA expression patterns and differential expression analysis in response to MLV and interferon treatments. (**A**) Violin plots showing FPKM distributions of lncRNAs, mRNAs, and TUCPs, revealing that mRNAs exhibit substantially higher expression levels compared to lncRNAs and TUCPs, which display low and narrowly distributed expression patterns. (**B**) Boxplots depicting FPKM distributions across four experimental groups (P1, P2, P3, and P4), demonstrating consistent median FPKM values across samples with minimal batch effects. (**C**) Heatmap of Pearson correlation coefficients among all samples, showing high pairwise correlations (R^2^ = 0.82–0.88) that confirm strong reproducibility and data quality. (**D**–**F**) Volcano plots illustrating differential lncRNA expression patterns in response to vaccination (P2 vs. P1, (**D**)), IFN-α1 treatment (P3 vs. P1, (**E**)), and IFN-ω5 treatment (P4 vs. P1, (**F**)). Upregulated lncRNAs are highlighted in red, downregulated lncRNAs in green, and non-significantly altered transcripts in blue. Progressive increases in differentially expressed lncRNAs are observed from the vaccine (442 up, 350 down) to the interferon-treated groups (IFN-α1: 799 up, 906 down; IFN-ω5: 1071 up, 1182 down), indicating that IFN-ω5 induces the most extensive transcriptional reprogramming in porcine alveolar macrophages. Abbreviations: FPKM, fragments per kilobase of transcript per million mapped reads; TUCP, transcripts of uncertain coding potential.

To quantify transcript expression, StringTie and Cuffdiff were used to calculate and evaluate FPKM values, which normalize for both sequencing depth and transcript length [24]. Figure 3A illustrates the global expression patterns of lncRNAs, mRNAs, and TUCPs (Transcripts of Uncertain Coding Potential). Detailed FPKM values for each transcript are provided in Supplementary Excel Table S3, which includes the complete list of novel and annotated porcine lncRNAs and mRNAs. Overall, mRNAs exhibited higher expression levels than lncRNAs and TUCPs, while both noncoding transcript types showed relatively low and narrowly distributed expression. This pattern aligns with the biological roles of lncRNAs, which typically act as fine-tuning regulators rather than high-abundance transcripts. As shown in Figure 3B, FPKM distributions across the four experimental groups (P1, P2, P3, and P4) were largely consistent, indicating uniform sequencing depth and reliable expression quantification among samples. The boxplots demonstrate that median FPKM values remained comparable across groups, suggesting minimal batch effects and robust data normalization. Additionally, the Pearson correlation (R^2^) analysis revealed high concordance among all samples (R^2^ = 0.82–0.88), confirming strong reproducibility and indicating that the RNA-seq data were of high quality and suitable for downstream differential expression analyses.

lncRNA expression responses to vaccination and interferon treatments were examined by performing differential expression analysis among the four experimental groups: P1 (control), P2 (vaccine), P3 (IFN-α1), and P4 (IFN-ω5). As shown in Figure 3D–F, the volcano plots illustrate the distribution of significantly upregulated and downregulated lncRNAs in each comparison relative to the control (P1). In total, 442 upregulated and 350 downregulated lncRNAs were identified in the vaccine group (P2 vs. P1), 799 upregulated and 906 downregulated in the IFN-α1 group (P3 vs. P1), and 1071 upregulated and 1182 downregulated in the IFN-ω5 group (P4 vs. P1). The proportion of DE lncRNAs increased from 8.25% in the vaccine group to 17.77% in the IFN-α1 group and peaked at 23.48% in the IFN-ω5 group, indicating that IFN-ω5 induces the most extensive transcriptional reprogramming. This increase was statistically supported by Fisher’s exact tests with BH correction, which yielded an odds ratio of 1.42 and a BH-adjusted p value of 1.49 × 10^−22^ when compared to the IFN-α1 group, and an odds ratio of 3.41 with a BH-adjusted p value of 3.86 × 10^−189^ when compared to the vaccine group. This trend suggests that lncRNAs may play essential regulatory roles in mediating host antiviral and immune responses following MLV vaccination and interferon stimulation. Consistent with our previous findings in microRNA and circRNA profiles, IFN-ω5 again exhibited the strongest ability to trigger broad immune and antiviral responses at the cellular level, highlighting its potent immunomodulatory effect in porcine macrophages.

To further visualize how IFN-responsive lncRNAs may connect with downstream gene programs, an lncRNA–mRNA interaction network was constructed using significantly altered transcripts (Figure S4). The resulting network displayed extensive connectivity, with a densely connected core and multiple peripheral subnetworks. Several highly connected hub transcripts were located at the center of the network, suggesting potential regulatory bottlenecks within the IFN-responsive transcriptional program. In contrast, smaller peripheral clusters likely represent more specialized gene sets. Overall, the network topology supports a coordinated regulatory structure during IFN-ω5 stimulation, in which a subset of lncRNAs may be centrally positioned to influence broader antiviral and inflammatory gene expression patterns.

**Figure 4:**
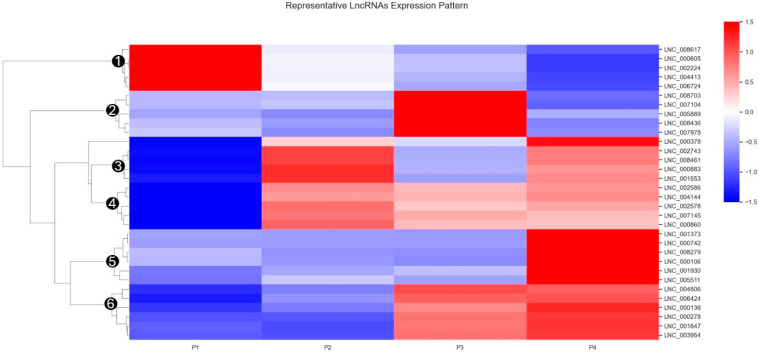
LncRNA expression values were normalized per transcript using Z-score transformation to facilitate cross-sample comparison. Hierarchical clustering was performed based on Euclidean distance and complete linkage to group lncRNAs exhibiting similar expression profiles across experimental conditions. Six major clusters (①–⑥) of lncRNAs are labeled on the dendrogram, each defined by distinct expression trends in the control (P1), vaccine (P2), and interferon-treated groups (P3: IFN-α1, P4: IFN-ω5). Expression levels are depicted using a blue-red gradient, with red indicating relatively higher expression and blue representing lower expression. This clustering analysis highlights dynamic and condition-specific regulation of lncRNAs in porcine alveolar macrophages following vaccination and interferon stimulation.

Differentially expressed lncRNAs were hierarchically clustered across all samples and visualized in Figure 4. For this analysis, each lncRNA’s expression profile was normalized using Z-score transformation to facilitate direct comparison among transcripts. Using Euclidean distance and complete linkage, clustering was performed to categorize lncRNAs with similar changes across experimental conditions. This approach identified six major clusters, each comprising lncRNAs with distinct and characteristic expression dynamics corresponding to the four treatment groups.

### 3.4. Identification of Interferon-Associated lncRNA–mRNA Co-Expression Modules and Regulatory Patterns

**Figure 5:**
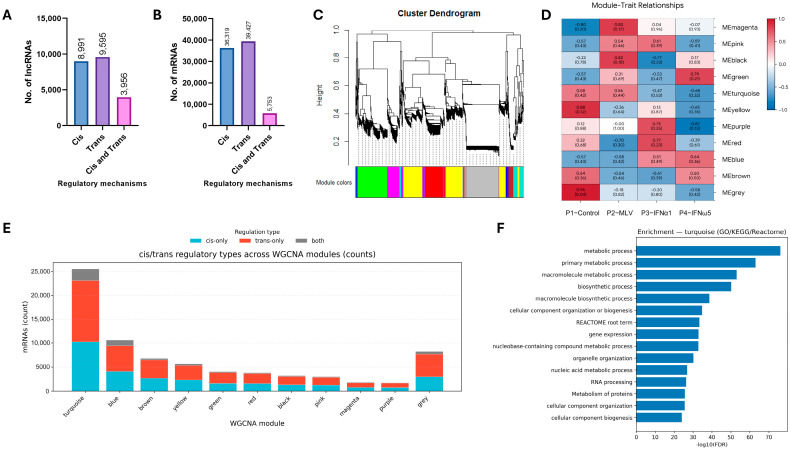
Cis/trans regulatory characteristics and WGCNA-based functional analysis of lncRNAs. (**A**,**B**) Numbers of lncRNAs (**A**) and mRNAs (**B**) predicted to participate in cis-, trans-, and combined cis–trans regulatory mechanisms. (**C**) Hierarchical clustering dendrogram of lncRNAs showing distinct WGCNA modules represented by different colors. (**D**) Module–trait correlation heatmap displaying relationships between module eigengenes and treatment groups (MLV, IFN-α1, IFN-ω5, and control). (**E**) Distribution of cis/trans regulatory types across WGCNA modules, illustrating module-specific regulatory patterns. (**F**) Functional enrichment of the turquoise module by GO, KEGG, and Reactome analyses, showing strong association with metabolic and biosynthetic processes.

As shown in Figure 5, the overall regulatory landscape of lncRNA–mRNA interactions was systematically characterized through network-based and enrichment analyses. The classification of predicted regulatory relationships indicated that trans-acting lncRNAs and their corresponding target mRNAs constituted the largest proportion, whereas predicted cis-acting and dual (cis and trans) regulatory types represented smaller fractions (Figure 5A,B). Hierarchical clustering of all expressed transcripts constructed by weighted gene co-expression network analysis (WGCNA) identified eleven distinct color-coded modules, each reflecting a unique co-expression pattern (Figure 5C). The module–trait correlation heatmap further demonstrated clear associations between module eigengenes and experimental treatments, with several modules showing strong positive correlations with IFN-α1 and IFN-ω5 stimulation (Figure 5D). Analysis of the regulatory composition across modules revealed that trans-only interactions predominated within major modules, particularly turquoise, blue, and grey (Figure 5E). Functional enrichment analysis of the turquoise module identified a significant over-representation of pathways related to metabolism, biosynthesis, gene expression, and cellular organization (Figure 5F). These findings outline the modular organization, regulatory type distribution, and functional characteristics of lncRNA–mRNA co-expression networks under interferon treatment.

### 3.5. Gene Ontology and KEGG Analysis of LncRNA-Targeted Genes

**Figure 6:**
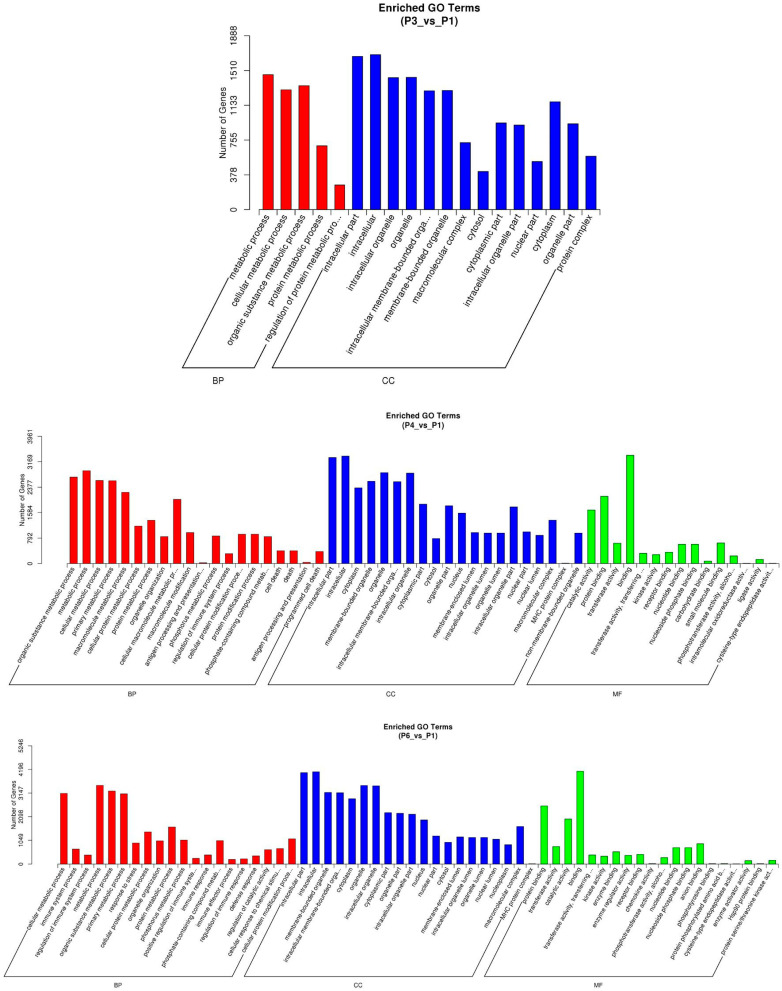
GO enrichment analysis was conducted for differentially expressed lncRNA-associated genes among treatment groups relative to the control (P1). The enriched GO terms were categorized into biological process (BP, red), cellular component (CC, blue), and molecular function (MF, green). Compared with the control, the vaccine group (P2 vs. P1) showed enrichment in metabolic and cellular processes; the IFN-α1 group (P3 vs. P1) displayed enrichment in immune and stress response–related terms; and the IFN-ω5 group (P4 vs. P1) exhibited broader enrichment involving protein binding, catalytic activity, and membrane-associated processes. These results indicate distinct functional regulation patterns of lncRNA-targeted genes under PRRSV infection and type I interferon stimulation. Some long terms are truncated with ellipses in the axis labels; their full names are provided here for clarity: cellular macromolecule metabolic process, antigen processing and presentation of peptide antigen, cellular protein modification process, phosphate-containing compound metabolic process, intracellular membrane-bounded organelle, transferase activity, transferring phosphorus-containing groups, phosphotransferase activity, alcohol group as acceptor, intramolecular oxidoreductase activity, interconverting aldoses and ketoses, cysteine-type endopeptidase activity involved in apoptotic signaling pathway, positive regulation of immune system process, cellular response to chemical stimulus, protein phosphorylated amino acid binding, and protein serine/threonine kinase activity, regulation of protein metabolic process.

**Figure 7:**
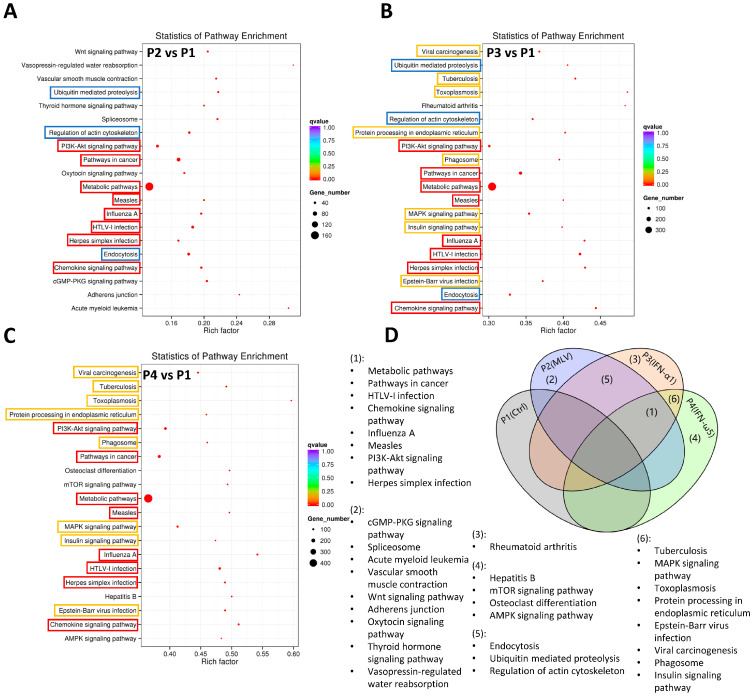
KEGG pathway enrichment analysis was conducted to identify signaling networks associated with differentially expressed lncRNAs between treatments and the control (P1). Scatter plots show the top 20 enriched pathways for (**A**) P2 vs. P1 (vaccine vs. control), (**B**) P3 vs. P1 (IFN-α1 vs. control), and (**C**) P4 vs. P1 (IFN-ω5 vs. control), ranked by q-value, rich factor, and gene number. Colored boxes indicate whether pathways are shared among multiple groups or unique to a specific treatment: blue highlights pathways shared by (**A**,**B**), orange highlights pathways shared by (**B**,**C**), and red highlights pathways shared by (**A**–**C**). The vaccine group was mainly enriched in metabolic and PI3K-Akt signaling pathways; the IFN-α1 group exhibited activation of chemokine, MAPK, and phagosome-related pathways; and the IFN-ω5 group displayed the broadest enrichment, including mTOR, AMPK, and ER protein processing pathways. (**D**) The Venn diagram summarizes shared and unique pathways among groups, highlighting overlapping immune-related pathways (PI3K-Akt, MAPK, chemokine) and IFN-ω5-specific engagement of broader metabolic and cellular regulatory networks. Abbreviations: AMPK, 5′ adenosine monophosphate-activated protein kinase; MAPK, mitogen-associated protein kinase; mTOR, mammalian target of rapamycin; PI3K-Akt, phosphoinositide 3-kinase-protein kinase B.

Gene Ontology (GO) enrichment analysis was performed to explore the potential biological functions of differentially expressed lncRNAs using the GOseq R package (v2.12), which corrects for gene length bias through the Wallenius non-central hypergeometric distribution [22]. The enriched GO terms were classified into three major domains: biological process (BP), cellular component (CC), and molecular function (MF). As shown in Figure 6, distinct enrichment patterns were observed among the treatment groups compared with the control (P1). In the vaccine group (P2 vs. P1), enriched terms were primarily associated with metabolic and cellular processes, suggesting general immune activation and metabolic adaptation following MLV vaccination. The IFN-α1 group (P3 vs. P1) displayed significant enrichment in categories related to immune response, response to stress, and intracellular organelles, indicating enhanced immune regulation and antiviral signaling. Notably, the IFN-ω5 group (P4 vs. P1) exhibited broader and stronger enrichment across all domains, particularly in protein binding, catalytic activity, and membrane-associated processes, implying that IFN-ω5 may be associated with a more complex transcriptional reprogramming involving both innate immunity and intracellular signaling pathways. Collectively, these findings suggest that type I IFN stimulation, especially IFN-ω5, promotes extensive regulation of lncRNA-associated genes implicated in antiviral defense and immune modulation in porcine alveolar macrophages. The biological processes and signaling networks associated with differentially expressed lncRNAs were further elucidated through KEGG pathway enrichment analysis, which was performed across six pairwise comparisons, with emphasis on P2 vs. P1 (vaccine vs. control), P3 vs. P1 (IFN-α1 vs. control), and P4 vs. P1 (IFN-ω5 vs. control)—all sharing the same baseline group (P1). The top 20 significantly enriched pathways were visualized as scatter plots, ranked by enrichment significance (q-value), gene number, and rich factor (Figure 7A–C).

In the vaccine group (P2 vs. P1), enriched pathways were primarily related to metabolic processes, PI3K-Akt signaling, and Influenza A infection, reflecting general immune activation and metabolic reprogramming following MLV vaccination. The IFN-α1 group (P3 vs. P1) exhibited broader enrichment involving chemokine signaling, phagosome formation, MAPK signaling, and viral infection–related pathways, consistent with enhanced antiviral and immune regulatory responses. The IFN-ω5 group (P4 vs. P1) showed the most extensive enrichment, with strong representation of mTOR, AMPK, and protein processing in endoplasmic reticulum pathways, highlighting its potent role in both metabolic regulation and immune signaling.

The Venn diagram (Figure 7D) summarizes shared and unique pathways among the treatment groups, showing overlapping activation of key antiviral and immune pathways—including PI3K-Akt, MAPK, and chemokine signaling—while IFN-ω5 uniquely engaged a wider range of cellular and metabolic processes. Consistent with our previous analyses of circRNAs and microRNAs, these findings further support that type I interferons, particularly IFN-ω5, induce more extensive transcriptional modulation through both immune signaling and metabolic adaptation pathways, underscoring their robust antiviral potential in porcine alveolar macrophages.

## 4. Discussion

Porcine reproductive and respiratory syndrome virus continues to impose a heavy burden on the global swine industry, causing severe economic losses worldwide [25]. Despite extensive research, an effective and broadly protective vaccine remains unavailable, underscoring the urgent need to clarify the underlying regulatory mechanisms and to develop novel antiviral strategies. Among host defense systems, non-coding RNAs (ncRNAs) have emerged as key regulators of antiviral responses to porcine viral infections, exhibiting diverse mechanisms of action [26]. Within this class, long non-coding RNAs stand out due to their remarkable stability, which is maintained through polyadenylation or secondary structures such as triple helices at their 3′ ends, as well as their pronounced tissue-specific expression patterns [26,27,28]. During viral infection, lncRNAs can function both in a cis manner, influencing adjacent genes within a limited genomic range, and in a trans manner, regulating the expression of distant genes and even genes located on different chromosomes. These multifunctional mechanisms enable lncRNA to coordinate complex cellular responses, such as interferon (IFN) signaling, apoptosis and metabolic reprogramming processes that are crucial for the antiviral defense of PAMs [12,29].

In this study, we profiled lncRNA expression in PAMs following stimulation with a PRRS modified live virus (MLV) vaccine and two type I interferons (IFN-α1 and IFN-ω5). The sequencing data captured the main transcriptional output of PAMs, with approximately 75% of reads mapping to protein-coding genes and ~1.5–1.9% to lncRNAs, providing sufficient depth to detect low-abundance transcripts (Figure S2). A stringent multi-step pipeline combining exon structure, transcript length, expression thresholds, and coding potential analysis (CPC, CNCI, and PFAM) refined 488,032 assembled transcripts to 9589 high-confidence lncRNAs. These exhibited typical structural features—shorter length, fewer exons, and smaller ORFs compared with mRNAs—and were predominantly classified as intronic, followed by intergenic and antisense types (Figure 1). Global expression analysis showed that lncRNAs were consistently expressed at lower levels than mRNAs, aligning with their regulatory rather than structural roles (Figure 2). Strong inter-sample correlations (R^2^ = 0.82–0.88) and uniform FPKM distributions confirmed high data quality and reproducibility (Figure 3). Differential expression analysis revealed a progressive activation pattern, with limited changes after vaccination but extensive modulation under interferon treatment—particularly IFN-ω5, which triggered the broadest transcriptional reprogramming. Hierarchical clustering identified six major expression clusters, reflecting distinct lncRNA responses linked to immune activation and interferon-specific antiviral regulation (Figure 4).

The composition and expression patterns of lncRNAs identified in this study are consistent with observations from infections caused by other members of the same viral family and related RNA viruses [30,31]. In human coronavirus infections, particularly SARS-CoV-2, lncRNAs have been shown to exert critical regulatory functions in controlling inflammation, immune dysregulation, and thrombosis through multiple molecular mechanisms. Key lncRNAs such as NEAT1, DANCR, MALAT1, C058791.1, TTTY15, and TPTEP1 were reported to modulate interferon signaling and the cytokine storm response, illustrating the reciprocal regulation between interferons and lncRNAs—whereby interferon signaling induces specific lncRNAs that, in turn, fine-tune antiviral gene expression [30,31].

In swine viral systems, similar regulatory paradigms have been observed. For instance, lncRNA446 was shown to restrict PEDV replication by inhibiting TRIM25-mediated ubiquitination and degradation of Alix, thereby maintaining tight junction integrity and epithelial barrier function [32]. Likewise, lncRNA HCG4 acts as a positive regulator of RIG-I signaling, enhancing K63-linked ubiquitination and IFN-β production to counteract the inhibitory effects of the influenza A virus NS1 protein. Collectively, these findings across diverse viral models highlight lncRNAs as conserved, dynamic modulators of interferon-mediated antiviral responses, contributing to both epithelial barrier protection and innate immune regulation [33].

To identify the key gene groups involved in regulatory control, the regulatory landscape of lncRNAs was further examined by constructing a weighted gene co-expression network (WGCNA) from the filtered expression matrix. Our findings align with previous observations that lncRNAs form co-expression modules enriched in immune and metabolic pathways, similar to the innate (yellow) and lung-specific immune (red) networks described by Lim et al. [34]. Eleven distinct co-expression modules were identified, each representing a cluster of highly correlated transcripts with shared regulatory features (Figure 5C,D). Correlation analysis revealed that the turquoise and grey modules were most strongly associated with IFN-ω5, while the red module correlated with IFN-α1 treatment, indicating interferon-specific transcriptional coordination. Consistent with earlier reports that both cis- and trans-acting interactions regulate interferon-related genes, our analysis revealed a clear predominance of trans-acting regulation, particularly within the turquoise, blue, and grey modules (Figure 5A,B,E). The red module, associated with IFN-α1, was linked to apoptosis and immune regulation, reflecting a focused antiviral response (Figure 5F). This suggests that interferon-inducible lncRNAs may primarily modulate distant gene targets, enabling the coordinated control of immune and metabolic pathways across the genome [34,35]. The turquoise module was enriched in processes related to macromolecule biosynthesis, gene expression, and metabolism, while the grey module involved protein folding and vesicle transport, implying roles in ER stress responses. Although our co-expression networks and pathway enrichment analyses provide a comprehensive overview of the regulatory landscape associated with IFN-ω5 stimulation, the functional roles of the identified lncRNAs remain putative. Although this study focuses on transcriptome-wide discovery, several high-priority lncRNAs within the IFN-ω5–associated turquoise module nevertheless emerged as promising candidates for further investigation. Their predicted involvement in antiviral, inflammatory, and metabolic pathways underscores the need for targeted functional assays. Future studies will focus on validating these lncRNAs using RT-qPCR in independent PAM samples and performing mechanistic experiments such as knockdown or overexpression to directly test their roles in IFN-mediated antiviral responses.

To further explore the hierarchical clustering patterns in Figure 4, the representative lncRNAs were mapped to their corresponding WGCNA module assignments. This analysis revealed strong concordance between the heatmap-defined clusters and the WGCNA module structure. Notably, Group 1 lncRNAs mapped exclusively to the blue module, and Group 4 lncRNAs mapped exclusively to the turquoise module, indicating that these clusters represent coherent co-expression programs. The remaining clusters were enriched for specific modules, including a green-dominant Group 5 and a brown-dominant Group 6, whereas Groups 2 and 3 showed mixed module membership but were enriched for magenta/green and red-associated lncRNAs, respectively. Together, these results strengthen the interpretation that the Figure 4 clustering patterns reflect coordinated transcriptional programs captured by WGCNA modules.

Functional enrichment analyses of predicted lncRNA target genes revealed treatment-specific biological signatures. The vaccine group was mainly enriched in metabolic and cellular processes, indicating moderate immune and metabolic activation. The IFN-α1 group showed enrichment in immune response and stress-related pathways, while IFN-ω5 induced the most extensive enrichment, spanning protein binding, catalytic activity, and membrane organization (Figure 6). KEGG pathway analysis supported these trends: the vaccine group activated PI3K-Akt and metabolic pathways, IFN-α1 engaged chemokine, MAPK, and phagosome signaling, and IFN-ω5 showed broad enrichment across mTOR, AMPK, and ER protein processing pathways (Figure 7). Shared pathways such as PI3K-Akt, MAPK, and chemokine signaling reflect core innate immune responses, whereas IFN-ω5-specific enrichment underscores its unique role in integrating immune signaling with metabolic adaptation. Previous work by Gao et al. in PRRSV-infected PAMs reported that differentially expressed lncRNAs were enriched in inflammation- and pathogen-induced pathways, including MAPK, cytokine–cytokine receptor interaction, and Jak-STAT signaling, providing strong precedent for our enrichment results [36]. Similarly, Peng et al. conducted integrative transcriptome profiling of PAMs during PRRSV infection and found that lncRNA-associated target genes were significantly enriched in interferon signaling, cytokine–cytokine receptor interaction, and antiviral pathways, including RIG-I-like receptor, Jak-STAT, and Toll-like receptor signaling [37]. Their KEGG analysis also revealed enrichment in PI3K-Akt, MAPK, and AMPK pathways, as well as metabolic processes linked to immune activation—closely paralleling the enrichment patterns observed in the IFN-ω5 group. Taken together, these comparisons suggest that lncRNAs act as key regulatory intermediaries in the interferon-driven transcriptional network that coordinates antiviral defense and cellular remodeling. These findings align with previous studies in which GO and KEGG enrichment analyses revealed that porcine lncRNAs are predominantly involved in interferon and cytokine signaling pathways, highlighting their active participation in innate immune and antiviral regulation [16,36]. Accordingly, these results indicate that lncRNAs contribute to host antiviral defense against PRRSV infection by modulating the expression of immune-related target genes in PAMs. The enrichment of immune-associated lncRNAs further suggests their involvement in coordinating cellular responses to antiviral signaling cues rather than direct viral infection. In addition, network analysis (Figure S4) provides systems-level support for the hypothesis that IFN-ω5–inducible lncRNAs participate in coordinated antiviral regulatory programs. The hub-centered topology and modular organization observed are consistent with a model in which a subset of IFN-ω5–responsive lncRNAs may act as key regulatory nodes linking IFN signaling to downstream inflammatory and antiviral gene expression. This network framework therefore helps prioritize highly connected lncRNAs for targeted functional validation in future studies.

Across our series of studies in PAMs treated with the vaccine strain or stimulated with IFN-α1 or IFN-ω5, a clear multi-layered noncoding RNA regulatory architecture emerges [5,18]. Among the three ncRNA classes, miRNAs display the smallest scale of differential modulation, whereas circRNAs exhibit a much broader transcriptional response, with more than one thousand circRNAs significantly altered across treatments. In the present study, lncRNAs exhibit the most extensive differential expression profile, with more than 2000 significantly differentially expressed transcripts under IFN stimulation. A pathway-level comparison of IFN-ω5 responses further highlights the distinct yet partially convergent regulatory roles of each ncRNA type. The lncRNA and circRNA datasets share only four pathways (Chemokine signaling, Epstein–Barr virus infection, Hepatitis B, and Pathways in cancer), while lncRNA and miRNA responses show a broader overlap of eight pathways, including key antiviral and stress-response pathways such as PI3K-Akt signaling, Metabolic pathways, AMPK signaling, Tuberculosis, Measles, HTLV-I infection, Herpes simplex infection, and Viral carcinogenesis. In contrast, circRNAs and miRNAs share only two pathways (Endocytosis and NF-κB signaling). This limited overlap is consistent with the mechanistic properties of circRNAs, which predominantly function through miRNA sponging and structural or stability-related regulation rather than broad transcriptional modulation, resulting in a narrower and more specialized pathway profile. Notably, no pathway is shared across all three ncRNA classes, indicating that each layer contributes a distinct regulatory dimension to the IFN-ω5 response.

Despite their differences, IFN-ω5 consistently emerges as the most potent inducer across all ncRNA categories. It generates the largest and most diverse set of enriched pathways for lncRNAs, circRNAs, and miRNAs, spanning antiviral, inflammatory, metabolic, and cancer-related signaling cascades. This broad activation suggests that IFN-ω5 induces a deeper and more comprehensive remodeling of cellular regulatory networks than either IFN-α1 or the vaccine treatment, and that its impact is propagated through multiple ncRNA mechanisms that are complementary rather than redundant. This observation is also consistent with our recent in vivo study using a replication-competent MLV vaccine expressing the IFN-ω5 to combat PRRSV, in which vaccinated pigs exhibited earlier immune activation, lower viremia and lung lesions, better weight gain, and overall protective effects comparable to those of licensed commercial MLV vaccines following PRRSV challenge [23,38,39].

Although the RNA-seq, co-expression, and enrichment analyses highlight several promising lncRNA candidates that may regulate interferon-mediated antiviral responses, direct functional validation remains technically challenging, particularly in PAMs, where transfection efficiency is low and stable overexpression systems are difficult to establish. We prioritized several lncRNAs for downstream mechanistic assays; however, practical constraints—including limited transfection tolerance, the lack of suitable expression vectors for long transcripts, and the low endogenous abundance of many lncRNAs—limited our ability to experimentally confirm their regulatory roles within the scope of this study. In addition, because a single defined PRRSV strain was used in this study, the extent to which these lncRNA signatures generalize across genetically diverse PRRSV strains remains to be determined. Therefore, the functional inferences presented here are primarily supported by computational predictions and co-expression patterns and will require targeted validation in future work. Future studies will also extend this analysis to additional PRRSV strains to provide a more comprehensive evaluation, potentially using antisense oligonucleotides, CRISPR-based modulation (CRISPRi/CRISPRa), or inducible expression systems in more tractable cellular models [5,18,40,41].

Overall, this study establishes a detailed molecular framework linking interferon signaling to lncRNA-driven transcriptional control in swine macrophages, offering new insights into host-targeted antiviral regulation. The comparative expression profiles further reveal both conserved and treatment-specific lncRNA signatures, suggesting that different interferon subtypes and vaccine stimulation activate distinct yet complementary lncRNA-mediated pathways that collectively modulate the innate immune response to PRRSV infection.

## Figures and Tables

**Table 1:** Summary of reads mapped to reference genome.

Sample Name	P1	P2	P3	P4
**Total reads**	88,711,244	81,698,196	83,905,840	90,253,536
**Total mapped**	77,138,322 (86.95%)	70,481,620 (86.27%)	74,412,691 (88.69%)	80,391,522 (89.07%)
**Multiple mapped**	3,705,473 (4.18%)	3,832,386 (4.69%)	3,838,200 (4.57%)	4,078,578 (4.52%)
**Uniquely mapped**	73,432,849 (82.78%)	66,649,234 (81.58%)	70,574,491 (84.11%)	76,312,944 (84.55%)
**Read-1**	36,746,731 (41.42%)	33,426,675 (40.91%)	35,279,472 (42.05%)	38,240,953 (42.37%)
**Read-2**	36,686,118 (41.35%)	33,222,559 (40.66%)	35,295,019 (42.07%)	38,071,991 (42.18%)
**Reads map to ‘+’**	36,693,528 (41.36%)	33,290,907 (40.75%)	35,265,462 (42.03%)	38,129,368 (42.25%)
**Reads map to ‘−’**	36,739,321 (41.41%)	33,358,327 (40.83%)	35,309,029 (42.08%)	38,183,576 (42.31%)
**Non-splice reads**	46,432,078 (52.34%)	42,026,077 (51.44%)	44,658,740 (53.22%)	48,409,573 (53.64%)
**Splice reads**	27,000,771 (30.44%)	24,623,157 (30.14%)	25,915,751 (30.89%)	27,903,371 (30.92%)
**Reads mapped in proper pairs**	70,997,620 (80.03%)	64,459,008 (78.9%)	68,346,900 (81.46%)	73,800,280 (81.77%)
**Proper-paired reads map to different chromosome**	0 (0%)	0 (0%)	0 (0%)	0 (0%)

## Data Availability

The raw sequencing data generated in this study have been deposited in the NCBI Sequence Read Archive (SRA) under BioProject accession number PRJNA882823.

## Supplementary Materials

The following supporting information can be downloaded at https://www.mdpi.com/article/10.3390/pathogens15010035/s1, Figure S1: The workflow of whole transcriptomic analysis (WTS) for lncRNA profiling in porcine alveolar macrophages; Figure S2: Distribution and classification of mapped reads across samples P1–P4; Figure S3: Chromosome-wide distribution of mapped read density (log2-transformed) across sense and antisense strands in PAM transcriptomes. Figure S4: LncRNA–mRNA interaction network constructed from STRING-based protein–protein interaction predictions; Table S1: Software used in the analysis; Table S2: Summary of quality control across four experimental groups; Table S3: Complete list of novel and annotated porcine lncRNAs and mRNAs with FPKM values.
