# Supplementary material for: Comparative Transcriptomics Reveals Novel and Differential Long-Noncoding RNA Responses Underlying Interferon-Mediated Antiviral Regulation in Porcine Alveolar Macrophages
Source: Pathogens. 2025 Dec 26;15(1):35. doi: 10.3390/pathogens15010035 (PMC12844857; doi:10.3390/pathogens15010035)
Supplement: Supplementary file 1 [file pathogens-15-00035-s001.zip › pathogens-4068075-supplementary/pathogens-4068075 - Supplementary 1/Supplemental Figure S1-S3.pdf]

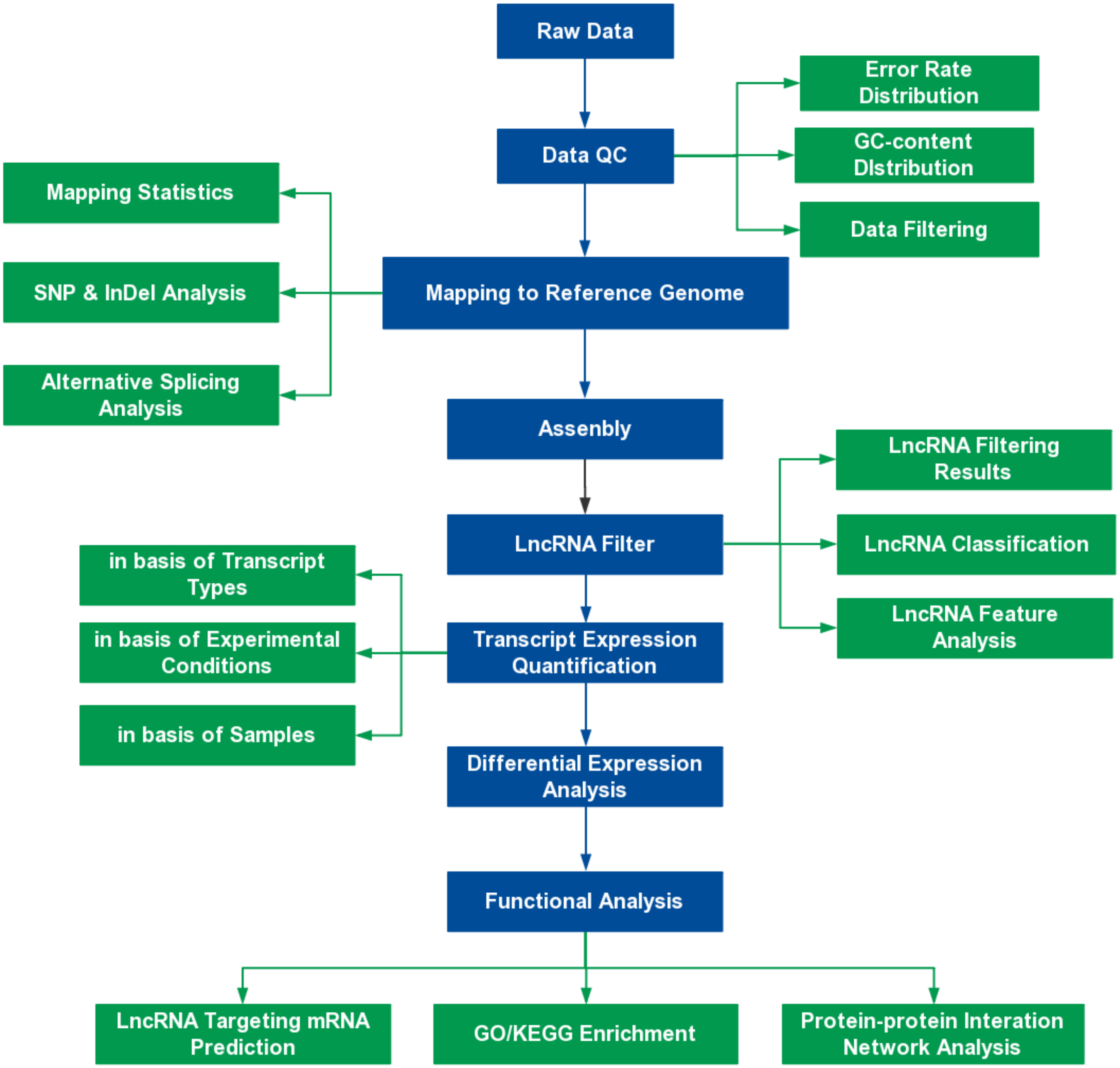

Figure S1: The workflow of whole transcriptomic analysis (WTS) for lncRNA profiling in porcine alveolar macrophages. After raw data quality control, filtered reads are mapped to the reference genome and assembled into transcripts. Candidate lncRNAs are filtered, classified, and characterized, followed by expression quantification and differential expression analysis. Functional analyses—including GO/KEGG enrichment, lncRNA target prediction, and protein–protein interaction network construction—are then performed to interpret the biological roles of the identified transcripts.

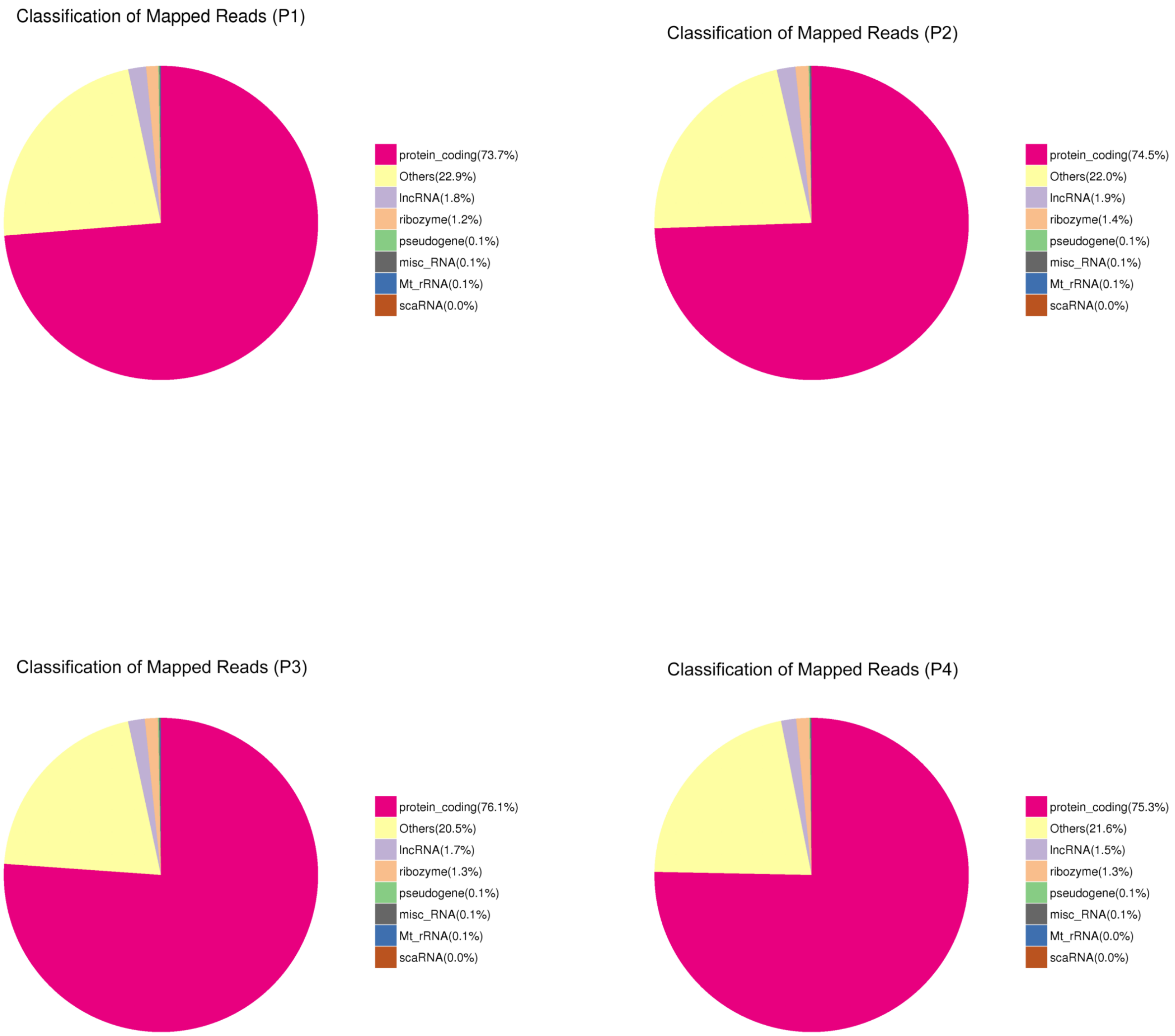

Figure S2: Distribution and classification of mapped reads across samples P1–P4.Pie charts show the distribution of mapped reads assigned to major transcript categories, including protein-coding genes, lncRNAs, rRNAs, pseudogenes, misc_RNAs, Mt_RNAs, and scaRNAs. Protein-coding reads constitute the majority in all samples (73–76%), followed by “Other” categories and lncRNAs (~1.4–1.9%). The patterns are consistent across all four samples, indicating comparable mapping and annotation quality.

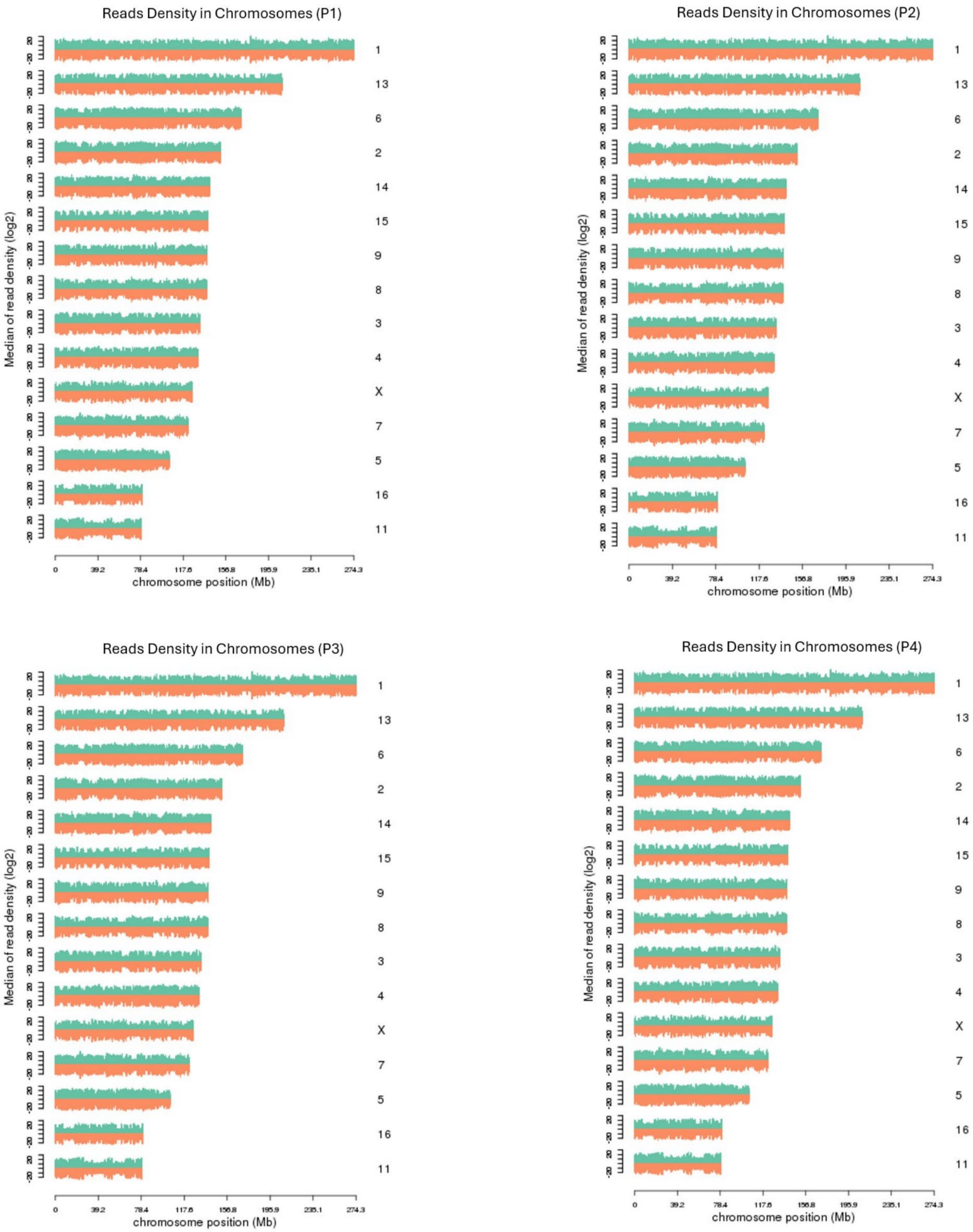

Figure S3: Chromosome-wide distribution of mapped read density (log_2-transformed) across sense and an-tisense strands in PAM transcriptomes. Chromosomes display consistent coverage patterns among samples, indicating comparable sequencing depth and mapping quality across the dataset.

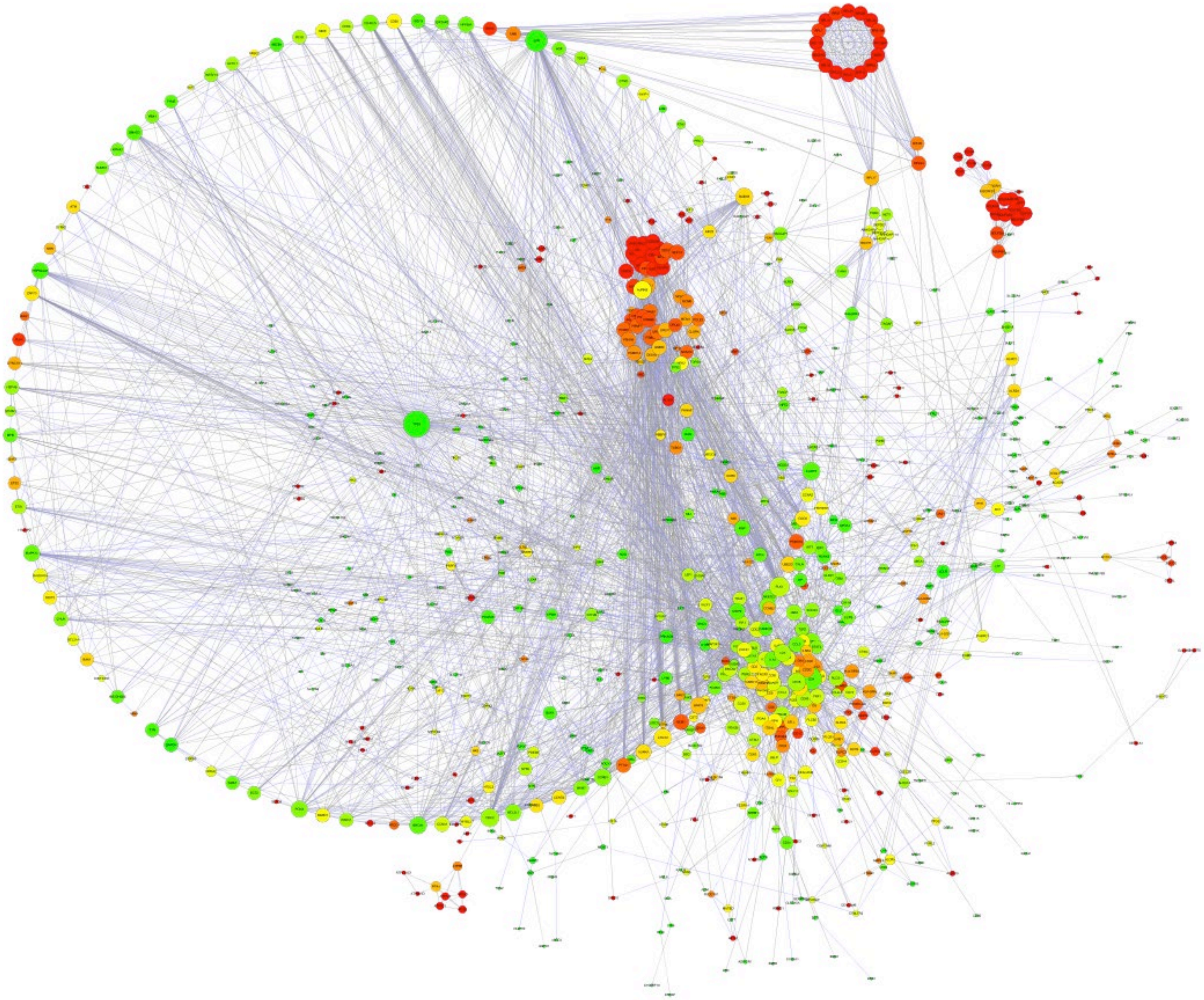

Figure S4: LncRNA–mRNA interaction network constructed from STRING-based protein–protein interaction predictions. For transcripts lacking direct annotation in STRING, BLASTx similarity searches were used to identify homologous genes in closely related species. The resulting PPI relationships were imported into Cytoscape for network construction and visualization. Nodes represent transcripts, and edges indicate predicted interaction links. Node size reflects degree (number of connections), with highly connected transcripts positioned centrally in the network. Node color indicates clustering coefficient, ranging from red (high connectivity) to green (low connectivity).
